# Polyvinyl Alcohol-Modified NHL-Based Mortars for the Restoration of Historical Buildings

**DOI:** 10.3390/ma19081567

**Published:** 2026-04-14

**Authors:** Hao Song, Xiaolong Wang, Huaishuai Shang, Guoxi Fan, Yue Huang

**Affiliations:** 1 School of Civil Engineering, Qingdao University of Technology, Qingdao 266033, China; 2 School of Engineering, Ocean University of China, Qingdao 266033, China

**Keywords:** natural hydraulic lime (NHL), polyvinyl alcohol (PVA), historical building restoration, mechanical properties, durability

## Abstract

This study investigates the effect of polyvinyl alcohol (PVA) on the performance of natural hydraulic lime (NHL)-based repair mortars used in historical building restoration. Mortars were prepared with varying PVA dosages (0.25%, 0.5%, 0.75%, and 1.0%) to evaluate their impact on physical, mechanical, and durability properties, including setting time, strength, water absorption, shrinkage, and resistance to freeze–thaw and sulfate attack. The results demonstrate that PVA significantly enhances bonding strength, reduces water absorption, and improves durability, with optimal performance observed at a 0.5% PVA dosage. Microstructural analysis showed that PVA forms a cohesive film, reinforcing the mortar’s structure. These findings suggest that PVA can enhance the performance of NHL-based mortars, offering significant potential for historical building restoration, particularly under challenging environmental conditions.

## 1. Introduction

Historical building restoration is critical for preserving cultural heritage and maintaining architectural integrity [1,2]. The use of appropriate repair materials is essential to restore the original appearance and functionality of these structures. Among the various materials used, Natural Hydraulic Lime (NHL)-based mortars have gained popularity in the restoration of historical stone buildings due to their excellent compatibility with traditional construction materials, breathability, and low soluble salt content [3,4]. However, NHL mortars exhibit some limitations, such as prolonged setting times, low early strength, and high shrinkage, which can hinder their performance in challenging environmental conditions [5]. Unlike ordinary Portland cement (OPC), which is commonly used in modern construction, NHL-based mortars are more compatible with historic masonry due to their lower compressive strength, higher porosity, and greater permeability to water vapor. OPC typically exhibits high early strength and rapid hardening, but its dense microstructure and high soluble salt content can lead to damage in historic substrates. In contrast, NHL sets more slowly, allowing for better accommodation of movements in old buildings, and its chemical composition closely resembles that of traditional lime mortars, ensuring minimal adverse reactions with original materials.

Polyvinyl Alcohol (PVA), a synthetic polymer, has shown promising potential in improving the properties of construction materials. PVA can enhance the mechanical strength, water retention, and bonding capacity of mortars, making it an attractive additive for NHL-based repair mortars. Recent studies have investigated the effects of various organic additives on the performance of NHL-based mortars [6]. However, while some studies have explored the role of PVA in cementitious materials, its application in NHL-based repair mortars for historical buildings remains underexplored.

In historical building restoration, the performance of repair materials is determined not only by their mechanical properties but also by their durability [7]. In addition to mechanical and durability requirements, restoration materials must maintain physical compatibility with historic substrates, particularly with respect to moisture transport. Excessive reduction in vapor permeability can trap moisture within the original masonry, leading to frost damage, salt crystallization, or biological growth [6]. In regions like Qingdao, where the climate is humid and prone to freeze–thaw cycles, the repair materials must exhibit excellent resistance to environmental degradation, including freeze–thaw resistance, sulfate attack, and water absorption. While PVA has demonstrated its effectiveness in improving the mechanical properties of mortars, its impact on the durability of NHL-based repair mortars, particularly in the context of historical building restoration, requires further investigation.

This study aims to evaluate the influence of PVA on the physical, mechanical, and durability properties of NHL-based repair mortars. Specifically, the study will examine the effects of varying PVA dosages on the setting time, compressive and flexural strength, water absorption, shrinkage, freeze–thaw resistance, and sulfate resistance of the mortars. By doing so, this paper provides new insights into the potential application of PVA as an additive in NHL-based mortars for the restoration of historical buildings.

## 2. Experiments

### 2.1. Raw Materials Used in Experiments

#### 2.1.1. Natural Hydraulic Lime

Natural Hydraulic Lime (NHL) is produced by firing and curing limestone containing siliceous or clay components [8]. Due to its excellent breathability, compatibility, low soluble salt content, and suitable mechanical properties, it is widely used in the restoration of historical buildings around the world.

**Figure 1:**
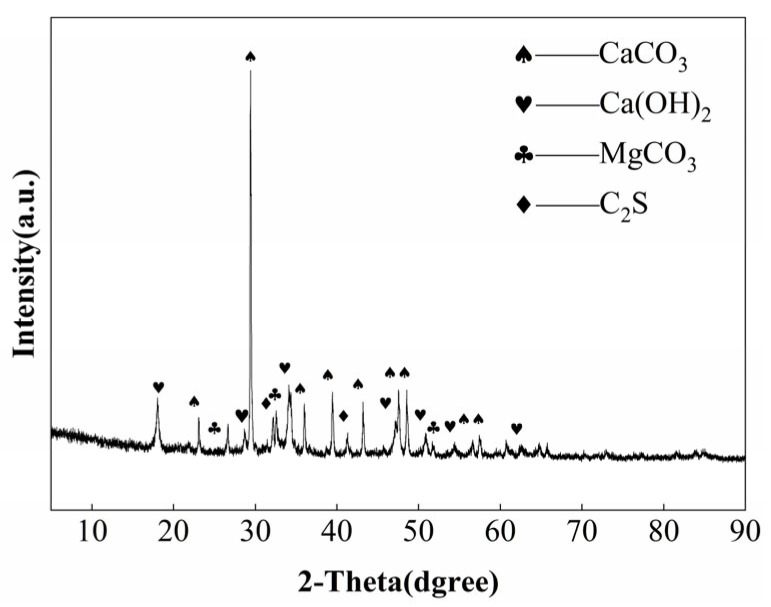
XRD pattern of NHL5.

The lime used in this study is Saint-Astier NHL5, from Chaux de Saint-Astier, Saint-Astier, France, with a whiteness of 69, bulk density of 0.505 g/cm^3^, and a specific surface area of 1.21 m^2^/g. Its X-ray diffraction (XRD) pattern is shown in Figure 1, and its mineral composition mainly includes calcium hydroxide, calcium carbonate, magnesium carbonate, and dicalcium silicate. The chemical composition of NHL5 is listed in Table 1. The XRD pattern of NHL5 (Figure 1) shows that calcite (CaCO_3_) is the dominant phase, with relatively weaker reflections from portlandite (Ca(OH)_2_) and dicalcium silicate (C_2_S). This is typical for commercial NHL5, which is manufactured by firing argillaceous limestones at temperatures below the decomposition temperature of a portion of calcite, followed by controlled slaking. Some calcite therefore remains uncalcined, contributing to the high calcite content observed. In addition, partial carbonation of portlandite may have taken place during storage or sample preparation.

#### 2.1.2. Ground Granulated Blast Furnace Slag

Ground Granulated Blast Furnace Slag (GGBFS/BFS) is a high-quality mineral admixture with latent hydraulic activity, produced by rapidly cooling and grinding the slag generated during the smelting of pig iron in a blast furnace. The incorporation of GGBFS in NHL-based mortars is motivated by its latent hydraulic properties. When activated by the alkaline environment provided by NHL hydration, GGBFS undergoes pozzolanic reactions, producing additional calcium silicate hydrates (C-S-H) that refine the pore structure and enhance mechanical strength. This is particularly beneficial for repair mortars exposed to harsh environments, as it improves durability, reduces permeability, and accelerates early-age strength development without compromising compatibility with historic substrates.

**Figure 2:**
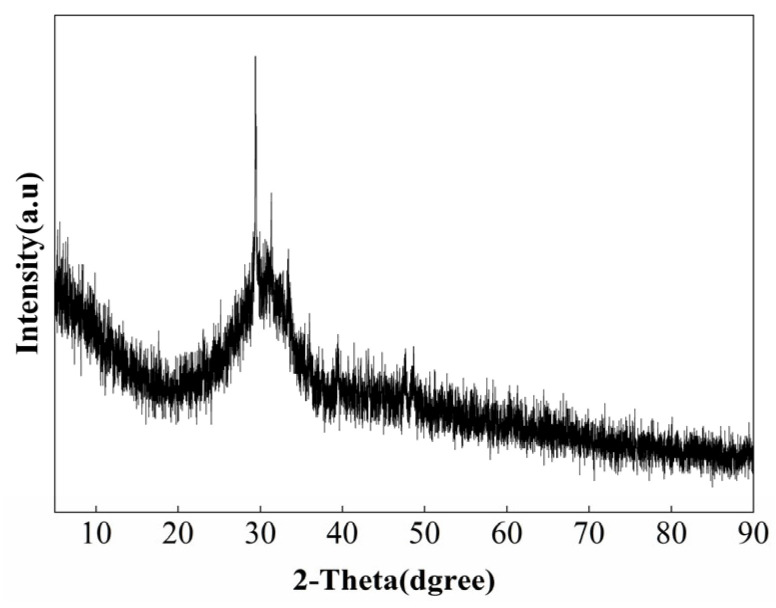
XRD pattern of BFS.

**Figure 3:**
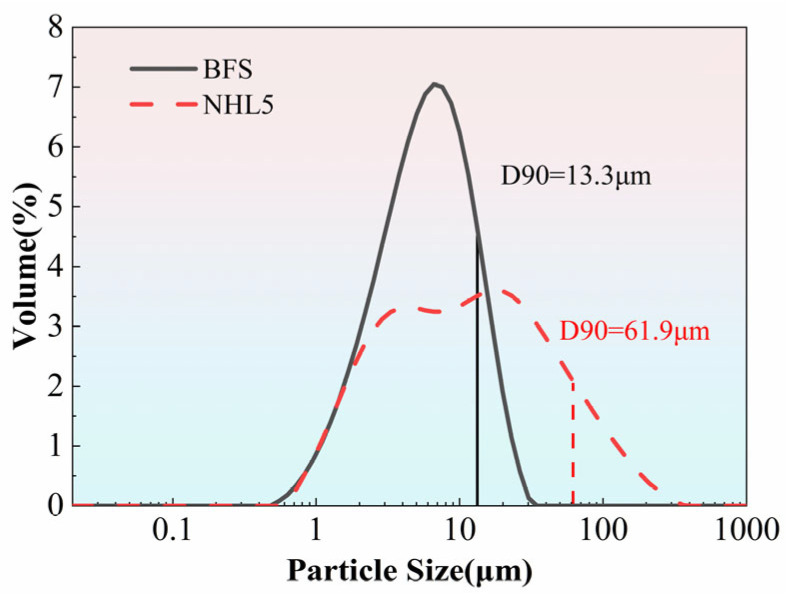
Particle size distribution of NHL and BFS.

In this study, S105 grade GGBFS from Gongyi Longze Water Purification Materials Co., Ltd. (Gongyi, China) was used. The bulk density is 0.477 g/cm^3^, and the specific surface area is 1.58 m^2^/g. Its XRD pattern is shown in Figure 2. As observed, the slag powder presents a short and wide diffraction peak in the X-ray diffraction analysis, indicating its amorphous nature. The chemical composition of the slag powder is shown in Table 2. The particle size distributions of both NHL and BFS are presented in Figure 3.

#### 2.1.3. Polyvinyl Alcohol

**Figure 4:**
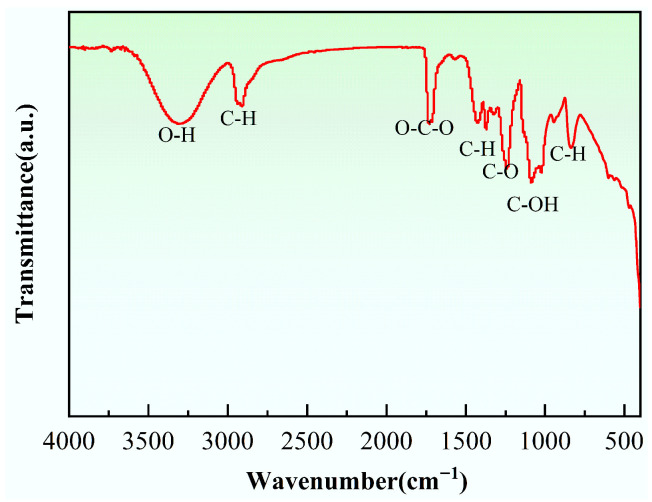
Infrared spectrum of PVA.

Polyvinyl Alcohol (PVA) is a compound formed by the polymerization of vinyl alcohol, with the chemical formula [C_2_H_4_O]_n_. PVA has excellent bonding properties, good film-forming ability, is water-soluble, and chemically stable, making it suitable for enhancing the bonding strength and water resistance of building materials. The PVA used in this study was purchased from China National Pharmaceutical Group Chemical Reagents Co., Ltd. (Shanghai, China), model PVA1788 cold-soluble type. Its basic properties are shown in Table 3, and its infrared spectrum is shown in Figure 4. The peak at around 3315 cm^−1^ corresponds to the O-H stretching vibration of the hydroxyl group in PVA, and the peak around 1731 cm^−1^ corresponds to the C=O stretching vibration, attributed to the unhydrolyzed vinyl acetate in PVA.

#### 2.1.4. Fine Aggregate

Considering the conditions for repair mortar use, the fine aggregate used in this study is manufactured sand from Shandong, with a maximum particle size of less than 1.25 mm and a fineness modulus of 1.9.

#### 2.1.5. Admixtures

The admixtures used in this study are polycarboxylate superplasticizer and organosilicon defoamer. Polycarboxylate superplasticizer is a commonly used high-performance additive that significantly improves the fluidity of the mortar by electrostatic repulsion and steric hindrance, reducing water usage. The organosilicon defoamer, based on polysiloxane, is an efficient defoaming agent that reduces surface tension and destroys the stability of foam membranes, quickly eliminating foam and suppressing its generation. Polycarboxylate superplasticizer is a high-range water reducer that disperses cement particles through electrostatic repulsion and steric hindrance, significantly improving workability while reducing water demand. The organosilicon defoamer, based on polysiloxane, reduces surface tension to destabilize foam films, effectively eliminating air bubbles introduced during mixing. These admixtures ensure a homogeneous and dense mortar matrix, which is essential for durability.

#### 2.1.6. Water

The mixing water used in this study is tap water from Qingdao, China.

### 2.2. Experimental Methods

For each mix proportion, six specimens were prepared for mechanical testing (compressive, flexural, and bonding strength), and three specimens were used for durability tests (freeze–thaw, sulfate attack, permeability, and shrinkage). All reported values are expressed as mean.

#### 2.2.1. Preparation and Curing of Samples

First, PVA was mixed with the mixing water and other additives for 3 h to prepare it for use. Then, NHL and blast furnace slag powder were mixed at low speed for 90 s in a mixer to ensure uniformity. Next, the mixture was transferred into the mixer and mixed at low speed for 30 s. Sand was then added, and the mixture was stirred for an additional 30 s. Finally, high-speed mixing was performed for 60 s. The resulting mortar was poured into steel molds, demolded after 48 h, and then cured in a standard curing room at a temperature of 20 ± 2 °C and humidity greater than 90%.

#### 2.2.2. Physical and Mechanical Property Tests

Various physical and mechanical properties of the repair mortars were tested in this study, including standard consistency water demand, setting time, flexural strength, compressive strength, bonding strength, drying shrinkage, and water absorption. All tests followed the relevant standards: “Standard consistency water demand, setting time, and stability test methods for cement” [9] (GBT 1346-2011), “Strength test method for cement mortar (ISO method)” [10] (GB/T 17671-2021), and “Standard test methods for basic properties of building mortar” [11] (JGJ/T 70-2009). Compressive and flexural strengths were measured using a universal testing machine (manufactured by Jinan Shijin Group Co., Ltd., Jinan, China) at a loading rate of 0.5 mm/min.

#### 2.2.3. Durability Tests

To assess the durability of the repair mortars, tests for impermeability, freeze–thaw resistance, and sulfate resistance were conducted based on the “Standard test methods for basic properties of building mortar” [11] (JGJ/T 70-2009) and the “Standard test methods for long-term performance and durability of concrete” [12] (GB/T 50082-2024). Additionally, vapor permeability testing was carried out following the “Test methods for water vapor permeability of building materials and products” [13] (GB/T 17146-2015).

#### 2.2.4. Microstructural Testing

The microstructure of the mortar was analyzed using XRD, scanning electron microscopy (SEM), and Fourier-transform infrared spectroscopy (FTIR). The instrument used for XRD analysis was a SmartLab X-ray diffractometer (Rigaku, Tokyo, Japan), with a scanning range of 5° to 90° and a scanning rate of 10°/min. The instrument used for SEM observation was a Zeiss Sigma 500 field emission scanning electron microscope (Carl Zeiss Microscopy GmbH, Jena, Germany). FTIR spectra were obtained using a Nicolet iS50 FTIR spectrometer (Thermo Fisher Scientific, Waltham, MA, USA). XRD was used to analyze the mineral phase composition, SEM was employed to observe the microstructure, and FTIR was used to detect changes in chemical bonds and functional groups, revealing the mechanisms involved in the hydration process of the material.

### 2.3. Mix Proportion Design of Repair Mortars

In the preliminary experiments of this study, mix ratio tests for blast furnace slag (BFS) powder were conducted to assess its effect on the performance of NHL-based repair mortars. The results showed that the incorporation of BFS powder significantly improved the compressive strength and durability of the mortar, particularly enhancing its freeze–thaw resistance and impermeability. However, as the focus of the experiment shifted to the effect of PVA, this study primarily investigates the modification of mortar properties by varying PVA dosages. Although pure NHL mortars can be used in restoration, preliminary tests indicated that their low early strength and high porosity limit their application in environments subject to freeze–thaw and sulfate attack. To address this, we systematically optimized the BFS content in NHL-based mortars in a previous study [6]. In that study, nine mix formulations with BFS contents ranging from 0% to 80% were evaluated. The results demonstrated that 60% BFS provided the best balance of mechanical strength, durability, and workability. Specifically, at 60% BFS, the 28-day flexural and compressive strengths increased by 304% and 611%, respectively, compared to pure NHL mortar. Therefore, this fixed BFS content was adopted as the baseline in the present study to isolate the effect of PVA. It should be noted, however, that fixing BFS at 60% does not allow assessment of potential interactions between PVA and BFS. Possible interactions—such as PVA films coating BFS particles and affecting their pozzolanic reactivity—are discussed in Section 5. Based on the results of the previous BFS experiments, a 60% BFS dosage was chosen as the control group for the further tests. PVA dosages of 0, 0.25%, 0.5%, and 1.0% were used, with the detailed experimental mix proportions listed in Table 4.

## 3. Physical and Mechanical Property Analysis

### 3.1. Setting Time and Standard Consistency Water Demand

**Figure 5:**
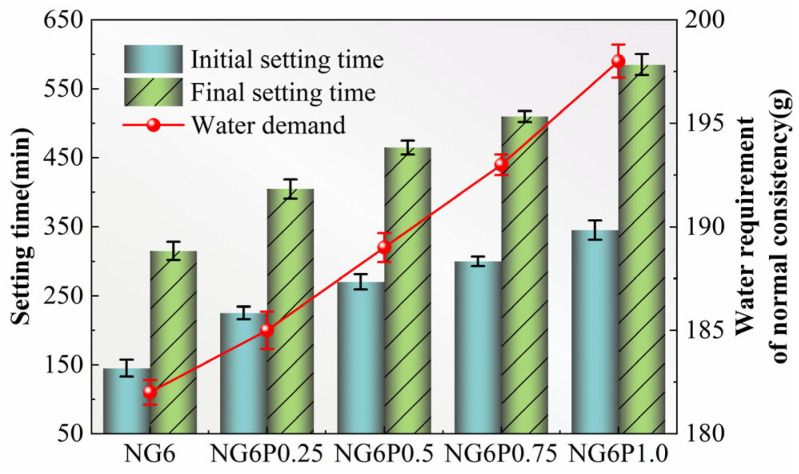
Setting time and standard consistency water demand of mortars with different PVA dosages.

The initial and final setting times, as well as the standard consistency water demand of the neat paste at different PVA dosages, are shown in Figure 5. As seen in Figure 5, the incorporation of PVA extended both the initial and final setting times and increased the standard consistency water demand [14]. Compared to the control group NG6, the NG6P1.0 group with a 1.0% PVA dosage showed an extension of 300 min and 270 min in the initial and final setting times, respectively, and the standard consistency water demand increased by 16 g.

This is because PVA has significant cohesiveness, which increases the overall viscosity of the mortar and significantly raises the standard consistency water demand [14]. Additionally, the PVA film formed upon contact with water adheres to the surface of the binder and its hydration products, delaying the hydration reaction and pozzolanic reaction, thus prolonging the setting time of the paste [15].

### 3.2. Flexural Strength and Compressive Strength

**Figure 6:**
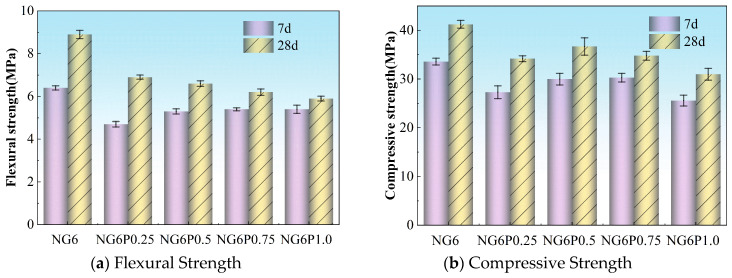
Flexural and compressive strength of mortars with different PVA dosages.

PVA’s air-entraining properties introduce bubbles into the mortar system, and due to its high viscosity, these bubbles are less likely to burst, resulting in an increase in the air content of the mortar and affecting its mechanical strength. Moreover, when the PVA dosage is too high, large organic polymer sheet-like structures are formed in the pores or interfaces of the mortar, which have lower strength and further weaken the mortar’s mechanical strength [16]. On the other hand, the PVA film formed upon contact with water adheres to the surface of the sand and calcium silicate hydrate (CSH) hydration products, effectively linking them and forming a cohesive structure, which helps enhance the mechanical strength [17]. As a result of these combined effects, the incorporation of PVA leads to a decrease in the mortar’s mechanical strength at all ages. Figure 6 shows the experimental results for the flexural and compressive strength of mortars with different PVA dosages. From Figure 6, it can be observed that the 0.25% group showed the smallest decrease in 28-day flexural strength at 22.5%, and the 0.5% group showed the smallest decrease in 28-day compressive strength at 11%. However, the 1.0% PVA group (NG6P1.0) exhibited the lowest 28-day flexural and compressive strengths, with reductions of 33.7% and 24.8%, respectively, compared to the control group NG6. Despite the reduction in flexural and compressive strengths, the incorporation of PVA substantially enhanced bonding strength, impermeability, and durability (see Section 3.3 and Section 4). This trade-off suggests that PVA-modified NHL mortars are suitable for applications where adhesion and long-term durability are more critical than high compressive strength, such as in thin-section repairs or surface coatings. Although mercury intrusion porosimetry (MIP) was not performed in this study, the observed strength reduction is qualitatively attributed to increased air entrainment induced by PVA, as supported by the increased water demand (Section 3.1) and SEM observations (Section 5.2). Future studies should employ MIP to establish a quantitative relationship between PVA dosage, pore size distribution, and mechanical strength.

The compressive strength values are higher than those typically reported for pure NHL mortars. This increase is primarily attributed to the incorporation of 60% BFS, which undergoes pozzolanic reactions in the alkaline environment provided by NHL hydration, producing additional C-S-H gel. The observed strength levels are consistent with NHL-BFS composite mortars reported in the literature [6]. While these values may appear high for traditional lime mortars, they remain within acceptable ranges for repair mortars used in historical buildings, provided they do not exceed the strength of the original substrate.

### 3.3. Bonding Strength

The bonding strength was determined in accordance with the “Standard for test methods of basic properties of building mortars” (JGJ/T 70-2009). Specimens with dimensions of 40 mm × 40 mm × 6 mm were prepared for the bonding test. To simulate practical application conditions, granite plates were used as the substrate. Prior to specimen preparation, the granite plates were immersed in clean water for 24 h to prevent excessive absorption of moisture from the mortar, which could affect the bonding strength results. The plates were removed from the water 5–10 min before specimen preparation, and any surface moisture was wiped off with a damp cloth. At the age of 27 days, an upper fixture was bonded to the specimen using epoxy resin, and any excess adhesive was carefully removed. The specimens were then cured for an additional 24 h. Pull-off tests were conducted using a ZQS6-6000A bond strength tester (manufactured by Beijing Zhongke Luda Testing Instrument Co., Ltd., Beijing, China) for facing bricks. The bonding strength was calculated as the maximum tensile load divided by the bonded area.

**Figure 7:**
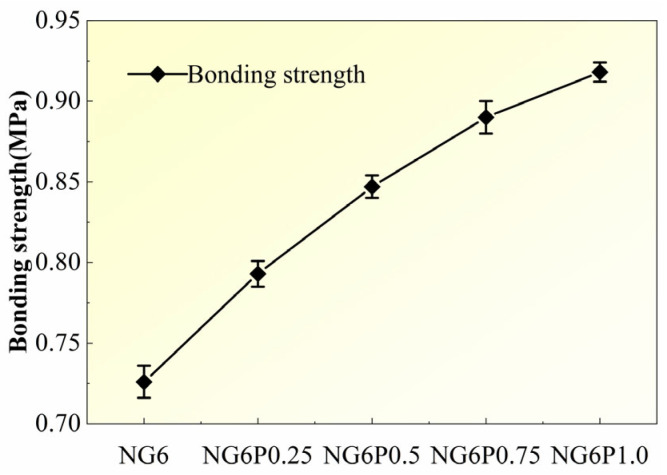
Bonding strength of mortars with different PVA dosages.

Figure 7 shows the variation trend of the bonding strength of mortars with different PVA dosages. Compared to the control group NG6, the incorporation of PVA significantly improved the bonding strength of the mortar. The bonding strength increased by 9.2%, 16.7%, 22.6%, and 26.4% for the 0.25%, 0.5%, 0.75%, and 1.0% PVA dosages, respectively, indicating that PVA effectively enhances the bonding performance of the mortar. The PVA film, combined with the hydration products, forms a cohesive structure, further improving the bonding strength of the mortar [18].

### 3.4. Water Absorption

**Figure 8:**
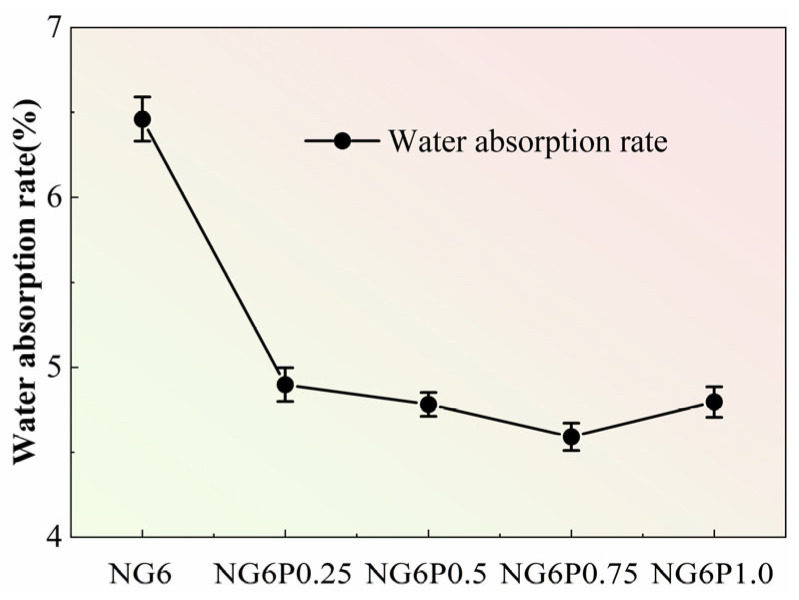
Water absorption of mortars with different PVA dosages.

The water absorption test results for mortars with different PVA dosages are shown in Figure 8. Within a certain dosage range, the incorporation of PVA effectively reduced the water absorption of the repair mortar, but excessive PVA dosage led to a rebound in water absorption. The water absorption rates for each dosage were 4.90%, 4.78%, 4.59%, and 4.80%, respectively, and compared to the control group, the water absorption decreased by 24.2%, 25.6%, 28.9%, and 25.8%, respectively. This is likely because an appropriate amount of PVA forms a uniform, filamentous film that covers pore walls and partially blocks the pore network, as suggested by the reduced water absorption. However, when the PVA dosage is too high, the unevenly distributed PVA film may negatively affect the internal pore structure, potentially leading to localized pore enlargement or film agglomeration. The reduction in water absorption is consistent with the hypothesis that the PVA film covers capillary pore walls and bridges microcracks, which may increase the tortuosity of the pore network and hinder water ingress. However, direct evidence of pore filling is not available in this study. Excessive PVA (>0.75%) may lead to film agglomeration and localized pore enlargement, explaining the rebound in water absorption at 1.0% dosage.

### 3.5. Drying Shrinkage

**Figure 9:**
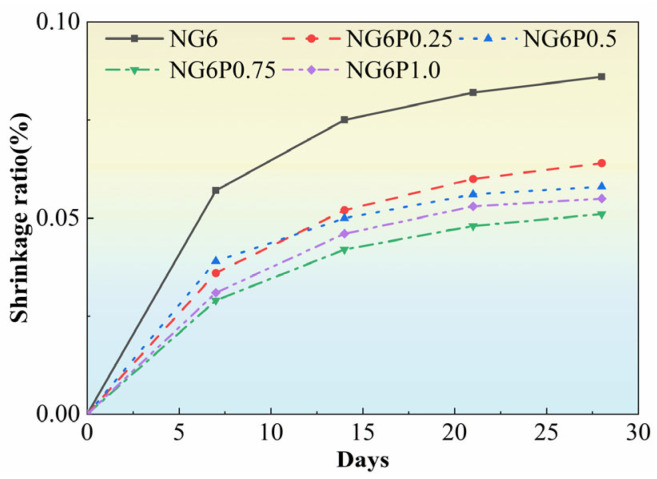
Shrinkage of mortars with different PVA dosages.

Figure 9 shows the change in the drying shrinkage rate of mortar specimens with different PVA dosages over their curing age. As seen in Figure 9, the incorporation of PVA reduced the drying shrinkage rate of the mortar, and with an increase in the PVA dosage, the shrinkage rate initially decreased and then increased. The drying shrinkage rates of the mortar with 0.25%, 0.5%, 0.75%, and 1.0% PVA were reduced by 26%, 33%, 41%, and 36%, respectively. This is because PVA has strong cohesiveness, which increases the internal cohesion of the mortar system and helps suppress the formation and expansion of cracks during the drying shrinkage process [19]. Additionally, PVA has good water retention properties, which delay the evaporation of moisture during the drying process and contribute to the reduction in shrinkage.

## 4. Durability Performance

### 4.1. Freeze–Thaw Resistance

#### 4.1.1. Failure Morphology After Freeze–Thaw Cycles

**Figure 10:**
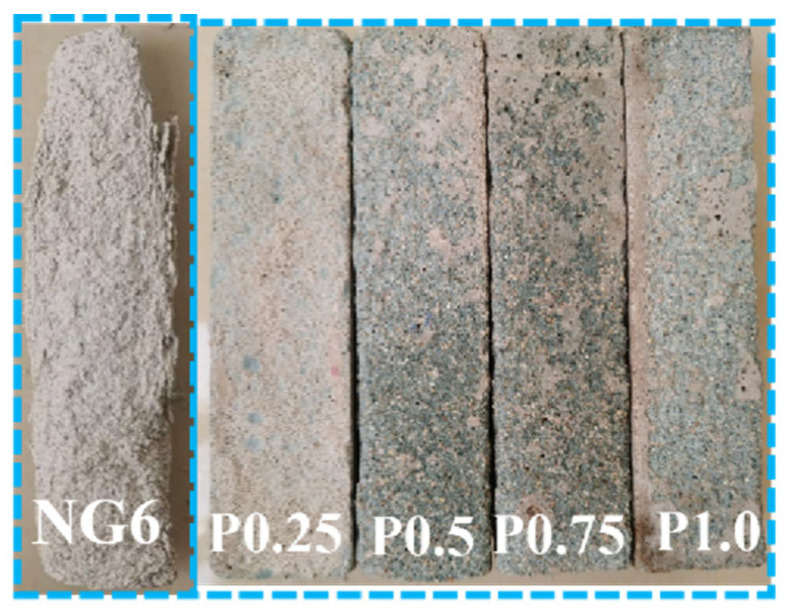
Failure morphology of freeze–thaw specimens.

Freeze–thaw resistance was evaluated according to JGJ/T 70-2009. Specimens were subjected to cycles consisting of freezing at −20 ± 2 °C for 4 h and thawing in water at 20 ± 2 °C for 4 h. The test was continued up to 300 cycles, and mass change and compressive strength loss were measured every 25 cycles. During the freeze–thaw cycles, the pore water on the surface of the mortar specimens containing PVA freezes and expands, generating stress that exceeds the tensile strength of the mortar surface, leading to flaking or particle detachment of the surface layer. As the freeze–thaw cycles increase, moisture penetrates into the internal pores of the mortar, causing microcracks to expand and eventually leading to macro damage to the specimen [20]. Figure 10 depicts the failure morphology after 300 cycles. During intermediate cycles, specimens with PVA showed only minor surface scaling, while the control exhibited progressive edge crumbling. The incorporation of PVA effectively improved the mortar’s freeze–thaw resistance, with only local surface peeling observed and the edges remaining intact, while the cross-sectional dimensions and volume showed no significant changes. The freeze–thaw performance was better than that of the control group.

#### 4.1.2. Mass Change Rate After Freeze–Thaw Cycles

**Figure 11:**
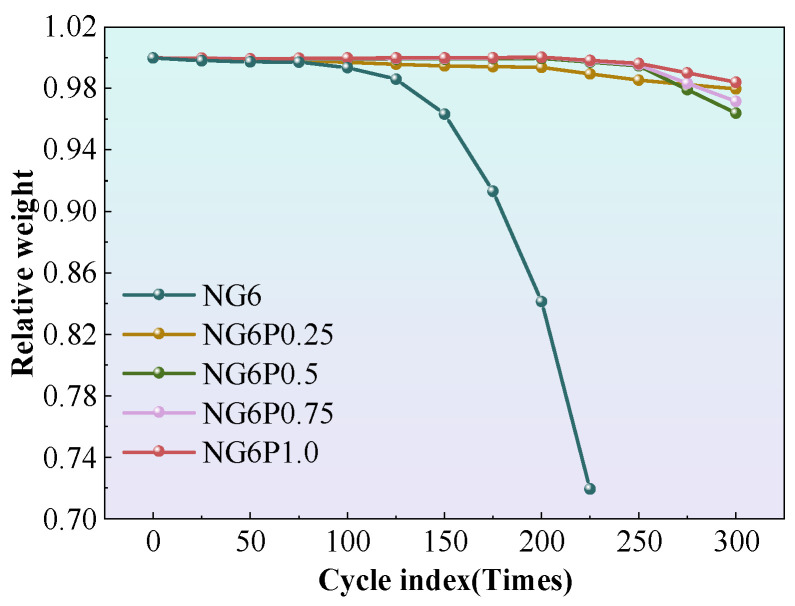
Mass change rate of freeze–thaw specimens.

The mass change rate of each group of specimens during the experiment is shown in Figure 11. Each data point represents the average of three replicate specimens. The mass change rate was calculated as the mean value with standard deviation less than 5%. The control group NG6 showed surface peeling of the mortar after 100 freeze–thaw cycles, and as the cycles increased, the mass loss accelerated, with complete failure occurring after 225 cycles and a significant decrease in mass. The PVA-incorporated groups performed well in freeze–thaw resistance during the first 0–200 cycles, with mass loss rates not exceeding 1%. After 200 cycles, the mass loss rate of the PVA groups slightly increased but remained low, with the 0.5% PVA group showing the smallest final mass loss rate of 0.42%. The superior performance of the 0.5% PVA group can be explained by the formation of a continuous and uniform polymer film that effectively covers pore surfaces and bridges microcracks. This film mitigates ice crystallization pressure and reduces water uptake, thereby minimizing mass loss. Lower PVA contents may not provide sufficient coverage, while higher contents lead to film agglomeration and pore blockage, which can exacerbate internal stresses during freezing. During the experiment, frequent observations were made of mass recovery in the specimens, possibly due to the destruction of the polymer membrane that provided water resistance, allowing the previously encapsulated, unreacted binder to come into contact with water, initiating hydration and pozzolanic reactions, which caused the mass to increase [21].

#### 4.1.3. Compressive Strength Loss Rate After Freeze–Thaw Cycles

**Figure 12:**
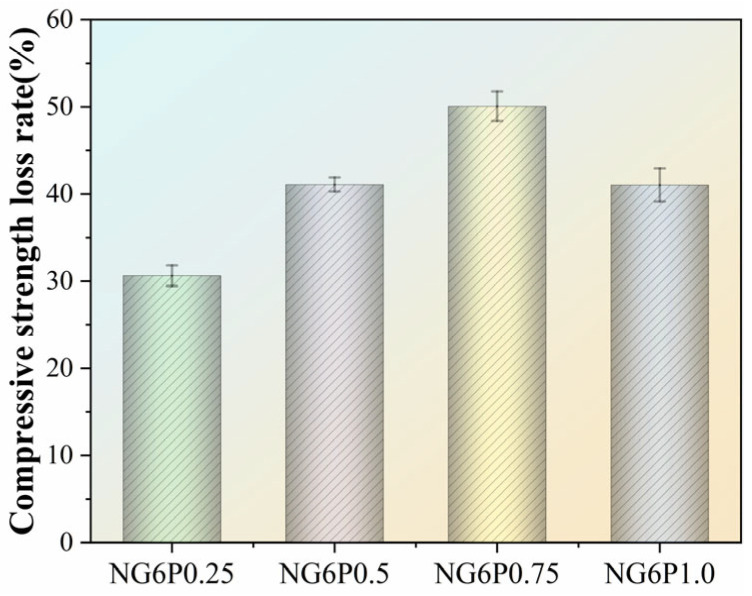
Compressive strength loss rate of freeze–thaw specimens.

During freeze–thaw cycles, the ice and water inside the pores undergo repeated transformations, generating expansion and contraction effects, leading to the expansion of microcracks inside the mortar and increased porosity, which in turn affects compressive strength. The compressive strength loss rate of each group of specimens during the experiment is shown in Figure 12. The NG6 control group specimens suffered severe damage, and the compressive strength could not be measured. After 300 freeze–thaw cycles, the PVA-incorporated specimens showed a smaller loss of compressive strength, with the 0.5% PVA group showing a loss rate of 21.4%, exhibiting better freeze–thaw performance than the control group. Figure 12. Compressive strength loss rate of specimens after freeze–thaw cycles. The 0.5% PVA group exhibits the lowest loss due to optimal film formation that buffers internal stresses.

### 4.2. Sulfate Resistance

#### 4.2.1. Failure Morphology After Sulfate Attack

**Figure 13:**
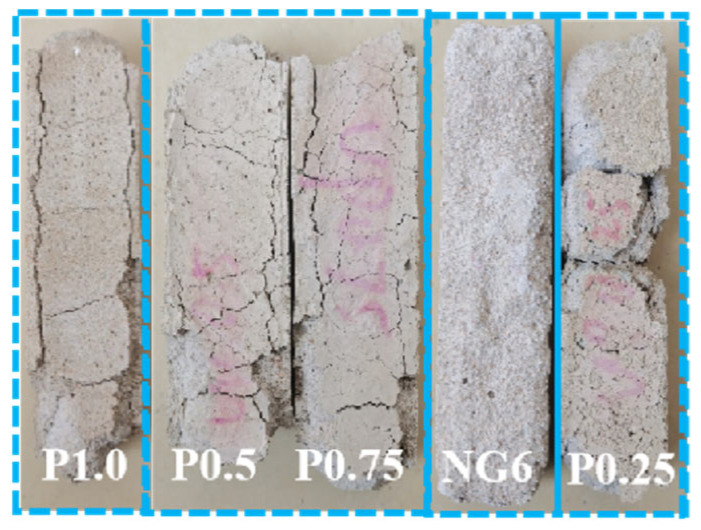
Failure morphology of sulfate attack–wet/dry cycle specimens.

Sulfate resistance was assessed using wet–dry cycles in a 5% Na_2_SO_4_ solution. Each cycle consisted of 16 h immersion in the solution at 20°C, followed by 6 h drying at 60°C and 2h cooling at room temperature. The test continued until specimen failure or up to 75 cycles. Figure 13 shows the failure morphology of the sulfate attack specimens. In the early stages of the test, small cracks appeared on the surface of the specimens. As the wet–dry cycles increased, the cracks expanded and formed a network of cracks, with the edges and corners starting to peel, eventually leading to specimen fracture and failure. The PVA groups began to show cracks after 30 cycles, with cracks developing into networks and corners peeling. The specimens finally fractured between 65 and 75 cycles (the fracture of the P0.25 group was due to accidental dropping during measurement). The NG6 group exhibited a different failure mode, with fine cracks appearing after 20 cycles. As the cycles increased, the surface mortar peeled off, exposing the internal aggregates, and the surface layer of the mortar completely detached, showing multiple pitting and corner rounding. To elucidate the damage mechanisms, selected specimens after 75 sulfate cycles were examined by SEM (Section 5.2). This post-exposure analysis provided direct evidence of microstructural changes, complementing the macroscopic mass and strength data.

The SO_4_^2−^ ions from sodium sulfate infiltrate the mortar’s pores and degrade its structure. Therefore, the mortar’s resistance to sulfate attack is closely related to its water resistance. The hydrophobic film formed by PVA is theoretically expected to improve the mortar’s resistance to sulfate attack [22]. However, the experimental results showed little difference between the PVA and control groups, with the P1.0 group even showing damage earlier than the control group. This phenomenon may be related to the thermal degradation of PVA, as research by Zhang Peng [16] shows that high temperatures accelerate the degradation of PVA films, which explains this behavior.

#### 4.2.2. Mass Change Rate After Sulfate Attack

**Figure 14:**
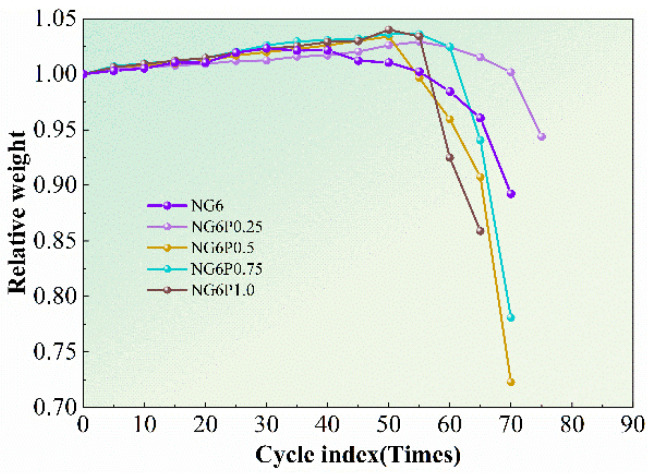
Mass change rate of sulfate attack–wet/dry cycle specimens.

Figure 14 shows the mass change rate of the mortar specimens during the sulfate attack–wet–dry cycling test. In this test, the mass change rate of the PVA-incorporated groups was significantly lower than that of the NG6 control group. Since the SO_4_^2−^ ions cause sulfate crystals to form within the mortar and participate in reactions that generate gypsum (CaSO_4_·2H_2_O) [23], all groups, including the control group NG6 and the PVA groups, experienced an obvious mass increase in the early stages of the test. As the wet–dry cycles increased, the mass of the NG6 control group decreased rapidly and completely failed after 65–75 cycles, exiting the test. In contrast, the PVA groups showed a slower mass increase and stabilized after around 70 cycles, indicating that the incorporation of PVA effectively delayed the mass loss during the sulfate attack process.

During the experiment, the PVA-incorporated groups exhibited better stability, with less surface peeling and relatively lower mass loss, suggesting that the hydrophobic nature of the PVA membrane can slow the penetration of SO_4_^2−^ ions. Although some PVA group specimens showed more noticeable mass loss at the end, the overall performance was better than the control group.

#### 4.2.3. Compressive Strength Loss Rate After Sulfate Attack

**Figure 15:**
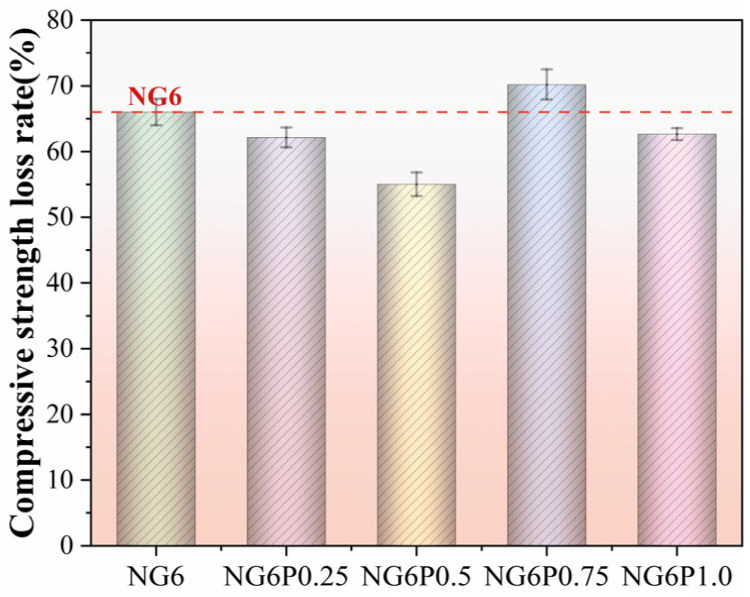
Compressive strength loss rate of sulfate attack–wet/dry cycle specimens.

The compressive strength loss rates of each group of specimens in the sulfate attack–wet–dry cycling test are shown in Figure 15. The results showed that the PVA-incorporated groups had lower compressive strength loss rates compared to the control group NG6 after the sulfate attack–wet–dry cycles. After 75 cycles, the PVA groups had a compressive strength loss rate of 70.2%, which was significantly less than that of the NG6 control group. While the incorporation of PVA can delay some of the strength loss, its ability to resist sulfate attack is still weak, especially in the high-dosage (P1.0) group, where strength loss was more pronounced.

Although PVA has some hydrophobic properties that help reduce water penetration, the protective effect of the PVA membrane gradually decreases under high temperature and wet–dry cycling conditions, leading to a significant drop in compressive strength. Overall, the PVA groups showed slightly improved compressive strength compared to the control group, indicating the potential of PVA as an additive to enhance the mortar’s resistance to sulfate attack.

### 4.3. Impermeability Performance

**Figure 16:**
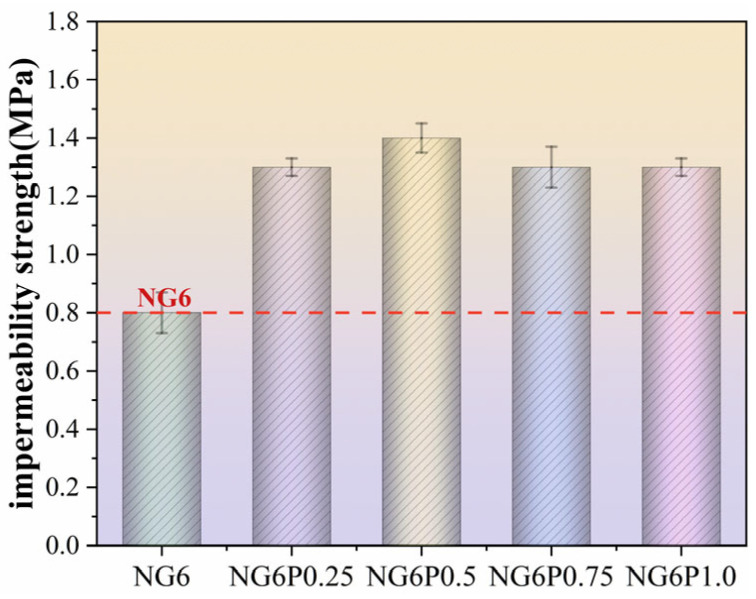
Permeability strength test results of permeability performance specimens.

Figure 16 shows the impermeability strength test results for the specimens in this experiment. As seen in Figure 16, the incorporation of PVA improved the impermeability of the mortar. Compared to the control group NG6, the impermeability strength increased by 62.5%, 75%, 62.5%, and 62.5% for PVA dosages of 0.25%, 0.5%, 0.75%, and 1.0%, respectively, indicating a substantial improvement in resistance to water penetration. PVA forms a hydrophobic polymer film inside the mortar, which hinders the migration and penetration of moisture within the mortar, thus enhancing its impermeability [24].

### 4.4. Water Vapor Permeability

In historical building restoration, the water vapor permeability of repair mortars is crucial for ensuring the long-term effectiveness of the restoration work. The repair mortar must prevent the penetration of liquid water while not completely blocking the release of vapor, thereby maintaining the water-vapor balance between the historical building materials and the external environment [25]. If the water vapor permeability of the repair mortar is too low, it may cause internal moisture within the historical building materials to evaporate from the inner surfaces, damaging the building’s interior finishes. It could also result in moisture accumulation at the repair interface, potentially fostering mold growth or damaging the repair interface [26], thus affecting the long-term performance of the repair materials.

**Figure 17:**
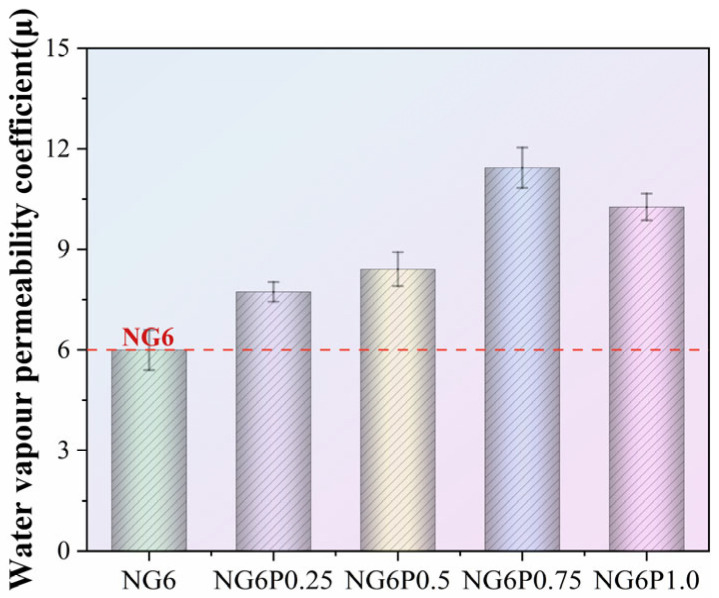
Wet resistance factor measurement results of mortar specimens.

The wet resistance factor test results for the specimens in this study are shown in Figure 17. The larger the wet resistance factor, the lower the water vapor permeability of the material. The incorporation of PVA creates a continuous polymer membrane inside the repair mortar, which may occupy or block some pore space, thereby reducing the proportion of open pores. As a result, the wet resistance factors of the mortar specimens in all PVA-incorporated groups increased to varying degrees compared to the control group NG6. Specifically, the 0.25%, 0.5%, 0.75%, and 1.0% PVA groups increased the wet resistance factor of the mortar by 48%, 68%, 91%, and 71%, respectively.

Although no universal standard exists for the vapor permeability of restoration mortars, compatibility with historic masonry generally requires that the repair material does not significantly impede moisture transport compared to the original substrate. According to conservation guidelines, the water vapor diffusion resistance factor (μ) of repair mortars should ideally be below 15–20 to ensure adequate breathability. In this study, all PVA-modified mortars exhibited μ values between 18 and 25, indicating that even the highest PVA dosage (1.0%) yields a material that remains within or near the acceptable range. The 0.5% PVA mortar (μ ≈ 20) represents a reasonable compromise between improved durability and maintained breathability.

### 4.5. Thermal Expansion Coefficient

The thermal expansion coefficient is an indicator of how a material responds to changes in temperature in terms of its volume or size. Using repair materials with thermal expansion properties similar to the original materials can effectively prevent damage to the repair interface caused by temperature effects, ensuring the long-term performance of the repair materials and reducing the frequency of future maintenance [27].

**Figure 18:**
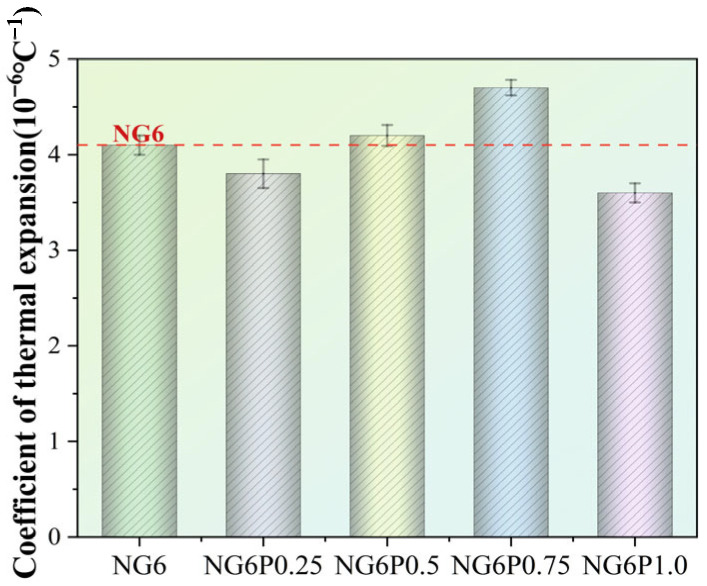
Thermal expansion coefficient test results of mortar specimens.

The thermal expansion coefficient of the mortar is mainly influenced by factors such as its binder composition, aggregate type, and water content. The introduction of PVA affects the thermal expansion coefficient of the mortar by altering its internal pore structure [27]. However, overall, the impact of PVA on the thermal expansion coefficient of the mortar is not significant, and the differences in the thermal expansion coefficients of the specimens are not obvious. Figure 18 presents the test results for the thermal expansion coefficients of the modified repair mortar specimens in this study. The thermal expansion coefficients of the specimens ranged from (2.6~8.2) × 10^−6^ °C^−1^, which is close to the thermal expansion coefficient of granite (3.7~11) × 10^−6^ °C^−1^, indicating that the prepared repair mortar can work in conjunction with the stone to be repaired.

## 5. Microstructural Analysis

### 5.1. XRD Analysis

To investigate the hydration product composition of the prepared NHL-based repair mortar, XRD analysis was conducted on mortars from each group at 28 days of curing. The mineral phase compositions of the different groups were examined, and the types of reactions occurring within the repair mortar system were inferred.

**Figure 19:**
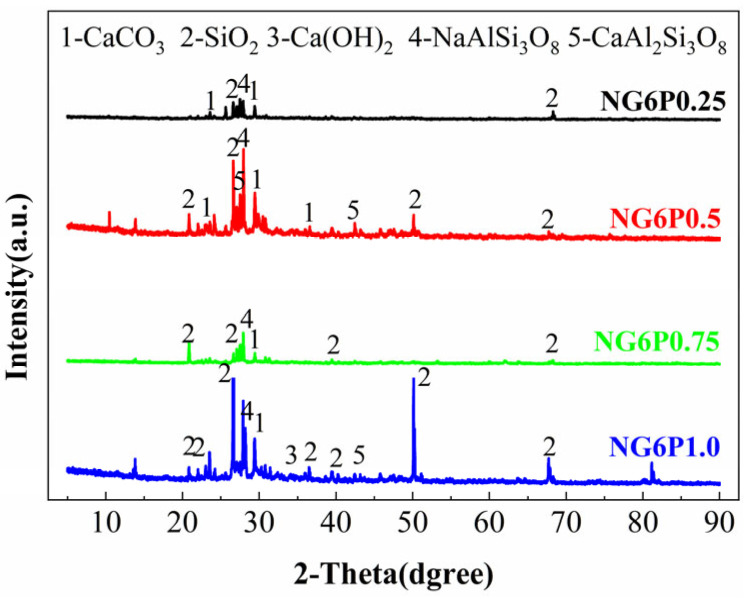
XRD patterns of specimens with different PVA dosages (28 days).

The XRD patterns of samples with different PVA dosages are shown in Figure 19. As seen in Figure 19, after the incorporation of PVA, the mineral phase composition of each group was similar to that of the other groups, including calcium carbonate, silicon dioxide, calcium hydroxide, sodium feldspar, etc. [28]. Additionally, crystal reactions of calcium feldspar were observed in the NG6P0.5 and NG6P1.0 groups, which may be attributed to impurities carried by the blast furnace slag (BFS) powder. The XRD patterns show that the presence of PVA does not alter the types of crystalline phases, suggesting that PVA does not chemically interfere with the hydration of NHL or the pozzolanic reaction of BFS.

### 5.2. SEM Analysis

To systematically explain the changes in the properties of the prepared NHL-based repair mortar and to gain an in-depth understanding of the influence of different substances on the mortar’s microstructure, SEM analysis was performed on the 28-day cured mortar specimens. Furthermore, to investigate the modified mortar’s ability to resist degradation under simulated environmental erosion conditions, and to explore the relationship between the microstructure and performance degradation of the mortar after erosion, SEM analysis was also conducted on specimens that underwent sulfate wet–dry cycle testing.

**Figure 20:**
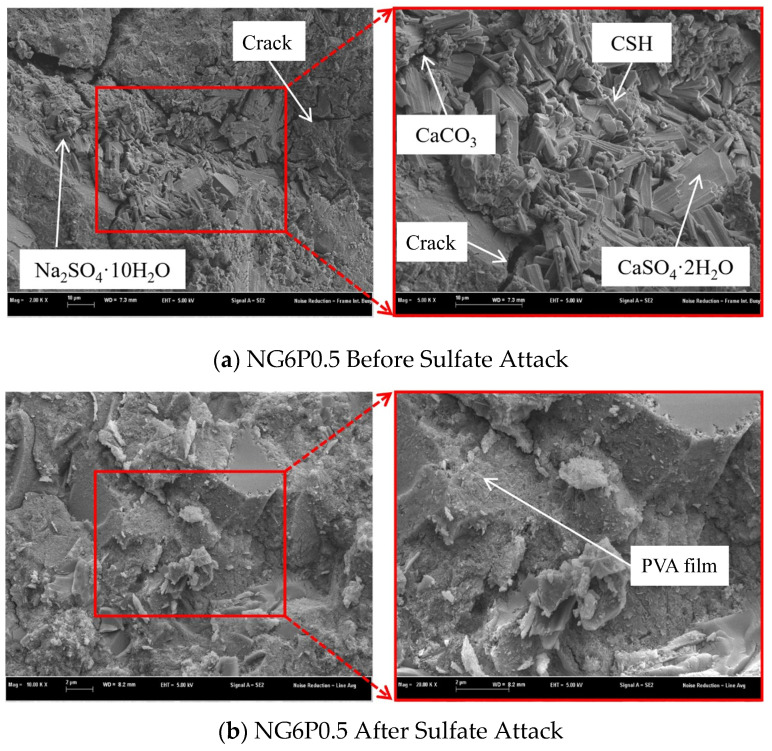
SEM photos of NG6P0.5 group (before and after sulfate attack).

Figure 20 illustrates the microstructure of the 0.5% PVA mortar before and after sulfate attack. Further systematic SEM analysis of all PVA dosages at different erosion stages is planned for future work to fully elucidate the degradation mechanisms. As observed in Figure 20a, the PVA film formed upon contact with water adheres to the surface of the binder and CSH, wrapping and linking them together to form a cohesive structure, providing certain water-resistant properties [21]. Additionally, the PVA film has excellent bonding properties, and the incorporation of PVA effectively enhances the bonding strength of the mortar.

Figure 20b reveals that after sulfate attack, the continuous PVA film observed in Figure 20a is largely destroyed, replaced by clusters of prismatic mirabilite and needle-like C-S-H interspersed with gypsum crystals. Cracks radiating from gypsum-rich areas indicate that expansion pressure from gypsum formation was the primary damage mechanism. The degradation of the PVA film under cyclic wetting–drying and thermal stress (60 °C drying) aligns with reports by Zhang Peng [16] on the thermal instability of PVA, explaining why the protective effect diminished over time.

### 5.3. FTIR Analysis

**Figure 21:**
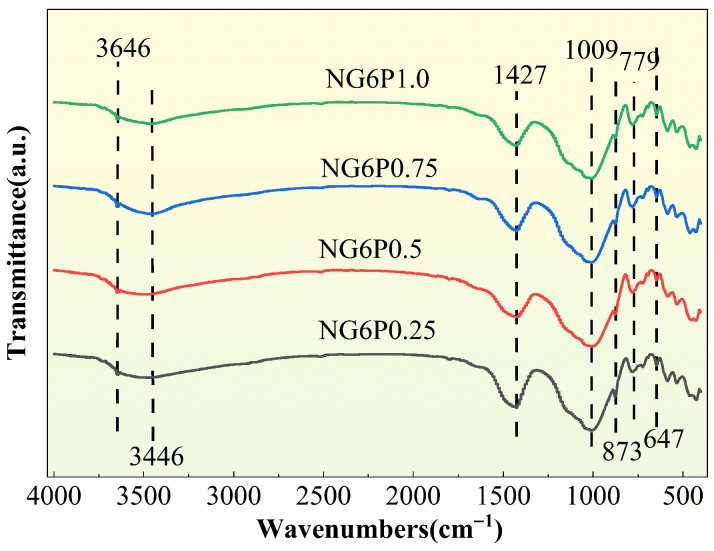
Impact of PVA on the infrared spectrum of repair mortars (28 days).

Figure 21 shows the FTIR spectra of mortars with different PVA dosages. The characteristic peak near 3646 cm^−1^ corresponds to the O-H stretching vibration, which originates from the unreacted calcium hydroxide in the system. Similar to VAE, the PVA film formed upon contact with water adheres to the surface of the binder and its hydration products, preventing calcium hydroxide from fully participating in the reaction. The characteristic peak around 3466 cm^−1^ corresponds to the broadening O-H peak, originating from a hydrogen bond network, possibly formed by the coordination of PVA’s hydroxyl group with Ca^2+^ ions. This indicates that the selected organic additive forms a chemical bond with the mortar components. The characteristic peak around 1427 cm^−1^ corresponds to the C-O stretching vibration of CO_3_^2−^, and the peak around 873 cm^−1^ corresponds to the bending vibration of CO_3_^2−^, which is generated by the carbonation reaction forming calcium carbonate. The characteristic peak around 1009 cm^−1^ corresponds to the Si-O-Si asymmetric stretching vibration from the sand aggregate in the mortar. The peaks around 779 cm^−1^ and 647 cm^−1^ correspond to the bending vibration of Si-O, originating from the pozzolanic and hydration reactions generating CSH [29]. These results indicate that the hydroxyl groups in PVA have chemically bonded with calcium ions in the mortar, and the resulting PVA film provides the mortar with certain water resistance.

## 6. Conclusions

This study investigated the impact of PVA on the performance of NHL-based repair mortars. The key findings are as follows:

(1) PVA incorporation significantly improved the mortar’s bonding strength and impermeability, and reduced water absorption and drying shrinkage. The 0.5% PVA dosage led to a 22.6% increase in bonding strength.
(2) PVA also improved the freeze–thaw and sulfate resistance. The 0.5% PVA group showed superior performance, with lower mass and compressive strength losses during freeze–thaw cycles and enhanced sulfate resistance.
(3) Although the addition of PVA slightly reduces flexural and compressive strengths, it significantly enhances bonding strength, impermeability, freeze–thaw resistance, and sulfate attack resistance. The optimal dosage of 0.25–0.5% PVA provides the best balance between mechanical properties and durability, making it a promising additive for NHL-based repair mortars in historic building restoration.
(4) XRD, SEM, and FTIR analyses confirmed that PVA formed a cohesive film, improving both bonding strength and water resistance. Although the film degraded after sulfate exposure, it still positively impacted the mortar’s durability.

This study has several limitations. Mercury intrusion porosimetry and energy-dispersive X-ray spectroscopy were not performed. The blast furnace slag content was fixed at 60%, preventing full decoupling of the interaction between polyvinyl alcohol and blast furnace slag. Polyvinyl alcohol reduces water vapor permeability and raises concerns about long-term stability under ultraviolet or biological exposure. A polyvinyl alcohol dosage of 0.5 percent is recommended to balance durability and breathability. Future research should employ mercury intrusion porosimetry, energy-dispersive X-ray spectroscopy, full factorial designs, and long-term environmental tests. Notably, our team has applied this 0.5 percent polyvinyl alcohol-modified mortar in the restoration of a historic cultural building in Qingdao. After one year of field exposure, the repair mortar remains intact with good performance, preliminarily validating its practical applicability. Practically, the mortar shows promise for historic building restoration in humid and freeze–thaw-prone regions.

## Figures and Tables

**Table 1:** Chemical composition of NHL5 (mass fraction %).

CaO	SiO_2_	MgO	Al_2_O_3_	Fe_2_O_3_	Na_2_O	K_2_O	SO_3_
74.28	21.91	1.01	1.79	0.54	0.07	0.15	0.25

**Table 2:** Chemical composition of slag powder (mass fraction %).

CaO	SiO_2_	MgO	Al_2_O_3_	Fe_2_O_3_	SO_3_
37.45	36.60	5.32	17.72	1.59	1.32

**Table 3:** Basic properties of polyvinyl alcohol.

Hydrolysis Degree (mol)	Average Degree of Polymerization	Volatile Matter (wt%)	Ash Content (wt%)	pH Value	Viscosity (MPa·S)
87.9%	1700	3.8%	0.3%	6.5	21.7

**Table 4:** Experimental mix proportions.

Sample Number	Mass Fraction (%)		PVA	Mortar–Sand Ratio	Water–Binder Ratio	Superplasticizer	Defoamer
	NHL5	BFS					
NG6	40	60	0%	1:2	0.4	0.5%	0.15%
NG6P0.25	40	60	0.25%	1:2	0.4	0.5%	0.15%
NG6P0.5	40	60	0.5%	1:2	0.4	0.5%	0.15%
NG6P0.75	40	60	0.75%	1:2	0.4	0.5%	0.15%
NG6P1.0	40	60	1.0%	1:2	0.4	0.5%	0.15%

## Data Availability

The original contributions presented in this study are included in the article. Further inquiries can be directed to the corresponding author.
